# Carbapenem-resistant *Enterobacterales* sepsis following endoscopic retrograde cholangiopancreatography: risk factors for 30-day all-cause mortality and the development of a nomogram based on a retrospective cohort

**DOI:** 10.1186/s13756-024-01441-1

**Published:** 2024-08-07

**Authors:** Hongchen Zhang, Yue Wang, Xiaochen Zhang, Chenshan Xu, Dongchao Xu, Hongzhang Shen, Hangbin Jin, Jianfeng Yang, Xiaofeng Zhang

**Affiliations:** 1 The Department of Gastroenterology, Affiliated Hangzhou First People’s Hospital, School of Medicine, Westlake University, No. 261 HuanSha Road, Zhejiang, China; 2 The Fourth School of Clinical Medicine, Zhejiang Chinese Medical University, Hangzhou First People’s Hospital, Hangzhou, 310003 China; 3 Key Laboratory of Integrated Traditional Chinese and Western Medicine for Biliary and Pancreatic Diseases of Zhejiang Province, Zhejiang, China; 4 Hangzhou Institute of Digestive Disease, Zhejiang, China

**Keywords:** Endoscopic retrograde cholangiopancreatography, Carbapenem-resistant *Enterobacterales*, Sepsis, Mortality, Nomogram

## Abstract

**Background:**

Endoscopic retrograde cholangiopancreatography (ERCP) has become a routine endoscopic procedure that is essential for diagnosing and managing various conditions, including gallstone extraction and the treatment of bile duct and pancreatic tumors. Despite its efficacy, post-ERCP infections – particularly those caused by carbapenem-resistant Enterobacterales (CRE) – present significant risks. These risks highlight the need for accurate predictive models to enhance postprocedural care, reduce the mortality risk associated with post-ERCP CRE sepsis, and improve patient outcomes in the context of increasing antibiotic resistance.

**Objective:**

This study aimed to examine the risk factors for 30-day mortality in patients with CRE sepsis following ERCP and to develop a nomogram for accurately predicting 30-day mortality risk.

**Methods:**

Data from 195 patients who experienced post-ERCP CRE sepsis between January 2010 and December 2022 were analyzed. Variable selection was optimized via the least absolute shrinkage and selection operator (LASSO) regression model. Multivariate logistic regression analysis was then employed to develop a predictive model, which was evaluated in terms of discrimination, calibration, and clinical utility. Internal validation was achieved through bootstrapping.

**Results:**

The nomogram included the following predictors: age > 80 years (hazard ratio [HR] 2.61), intensive care unit (ICU) admission within 90 days prior to ERCP (HR 2.64), hypoproteinemia (HR 4.55), quick Pitt bacteremia score ≥ 2 (HR 2.61), post-ERCP pancreatitis (HR 2.52), inappropriate empirical therapy (HR 3.48), delayed definitive therapy (HR 2.64), and short treatment duration (< 10 days) (HR 5.03). The model demonstrated strong discrimination and calibration.

**Conclusions:**

This study identified significant risk factors associated with 30-day mortality in patients with post-ERCP CRE sepsis and developed a nomogram to accurately predict this risk. This tool enables healthcare practitioners to provide personalized risk assessments and promptly administer appropriate therapies against CRE, thereby reducing mortality rates.

## Introduction

Endoscopic retrograde cholangiopancreatography (ERCP), which was first performed in 1968, has become a routinely performed endoscopic procedure that has proven to be effective in diagnosing and treating various conditions, including gallstone removal and bile duct and pancreatic tumor treatment [1]. ERCP is the gold-standard therapeutic modality for treating diseases affecting the biliary and pancreatic ducts. The prevalence of post-ERCP infections is less than 5% [2]. High hygienic standards during the procedure, along with proper disinfection and storage of endoscopic equipment, have significantly reduced infection rates. However, failure to reestablish drainage after the infusion of contrast media into obstructed bile ducts during ERCP remains the primary risk factor for post-ERCP infections [3]. Post-ERCP infections pose a significant danger and could potentially lead to life-threatening sepsis, particularly when these infections are associated with carbapenem-resistant *Enterobacterales* (CRE) [4].

CRE comprises gram-negative bacteria that are resistant to carbapenem antibiotics, which are often considered the last line of defense against multidrug-resistant infections [5]. CRE infections are a major concern because of their resistance to carbapenems and other antibiotics, thus leading to fewer effective therapeutic options. Predominant CRE types include *Klebsiella pneumoniae* and *Escherichia coli*. High antibiotic resistance in CRE leads to increased treatment failure, extended hospitalizations, increased healthcare costs, and significantly elevated mortality rates [6]. A study in a Thai tertiary care institution reported an in-hospital mortality rate of 68.33% among CRE-infected patients [7].

Post-ERCP infections represent a substantial clinical hurdle, with the etiological spectrum encompassing a diverse array of microbial entities [8]. The connection between ERCP and subsequent infections is often attributed to the procedural disturbance of innate infection barriers in the biliary and pancreatic ductal systems, thus creating a route for microbial invasion. The clinical manifestations of post-ERCP infections range from mild cholangitis to severe sepsis, significantly increasing the complexity of patient management and disease prognostication. The emergence of CRE as a predominant pathogen in post-ERCP infections heralds a daunting clinical scenario [9]. Post-ERCP sepsis is an acute-onset infection that often has a poor prognosis due to the limited availability of successful antimicrobial treatments. The complex relationship between the post-ERCP anatomical milieu and CRE pathogenicity mandates a thorough exploration of the prognostic determinants governing the clinical course of post-ERCP CRE sepsis. Numerous studies have explored prognostic models for patients with CRE infections or similar conditions with the aim of predicting 30-day mortality [10, 11]. Nonetheless, a notable gap persists in the literature concerning patient-centered predictive paradigms specifically tailored for post-ERCP CRE sepsis.

This study aimed to identify the risk factors for 30-day mortality in patients with CRE sepsis following ERCP and to develop and validate a nomogram that can be used to accurately predict 30-day mortality risk. By combining several important prognostic factors into a simple graphical tool, this nomogram will help clinicians assess mortality risk quickly.

## Subjects and methods

### Study design and subjects

This retrospective analysis examined the clinical data of patients who underwent inpatient ERCP at the Department of Gastroenterology, Affiliated Hangzhou First People’s Hospital, School of Medicine, Westlake University, from January 2010 to December 2022. Detailed records of the demographic and clinical characteristics of these individuals were kept. The inclusion criteria were patients who exhibited sepsis and positive CRE blood culture results within 5 days post ERCP. The exclusion criteria were as follows: (1) patients for whom essential information was lacking; (2) individuals displaying any signs of bacteremia before ERCP, including symptoms or abnormal laboratory results; (3) patients who were given antibiotics before ERCP; (4) patients with a confirmed infection in other areas, such as pneumonia or urinary tract infection after ERCP; and (5) individuals younger than 18 years. This study utilized a retrospective cohort design. The primary outcome was the mortality rate within one month after the first positive blood culture for CRE. The survivor and nonsurvivor subgroups were analyzed together to determine the predictors of mortality. The survival data were analyzed via a Cox regression model to identify risk factors, which was useful for the development of a predictive model. This model was then used to develop a nomogram to assess the 30-day mortality rate for patients with post-ERCP CRE sepsis.

Approval for the research protocol was obtained from the Research Ethics Committee (ZN20231106) of the institution. Due to the retrospective nature of the analysis, the requirement to obtain written informed consent was waived.

### Clinical and epidemiological data

The following data were extracted from medical records: patient characteristics (age, sex, and Charlson comorbidity index); exposures in the 90-day period before ERCP (use of antibiotics, hospitalization, invasive procedures, and intensive care unit [ICU] admission); exposures in the 30-day period before ERCP (use of immunosuppressive drugs); epidemiological information (time interval from ERCP to the onset of CRE sepsis); presence of comorbid conditions (previous infection with CRE, cerebrovascular diseases, malignant tumors, diabetes, cirrhosis, and hypoproteinemia); severity of illness at the time of CRE sepsis onset (quick Pitt bacteremia score and Acute Physiology and Chronic Health Evaluation [APACHE] II score); reasons for performing ERCP (malignant biliary stricture, benign biliary stricture, bile duct stone, pancreatic duct stone, pancreatic duct stricture, bile leak, and pancreatic fistula); details related to the procedure (placement of biliary stent, cholangioscopy, biliary sphincterotomy, removal of bile duct stone, bile duct radiofrequency ablation, total duration exceeding 45 min, occurrence of post-ERCP pancreatitis, post-ERCP perforation, and post-ERCP bleeding); and management of antibiotic therapy (inappropriate initial treatment, delayed definitive treatment, and short treatment duration [therapy lasting less than 10 days]). The main focus of the study was to examine the risk of all-cause mortality within a period of 30 days.

### Definitions

We defined CRE sepsis as a bloodstream infection confirmed by the presence of a CRE strain in blood culture, along with a Sequential Organ Failure Assessment (SOFA) score of ≥ 2, according to the Sepsis 3.0 guidelines [12]. Before a susceptibility report is available, empirical therapy involves administering antimicrobials. Appropriate empirical therapy was defined as the administration of in vitro active antimicrobials against the isolates within 24 h of infection onset, which continued for at least 48 h [13]. Treatments that did not meet these requirements were considered inappropriate. The administration of antimicrobial treatment after susceptibility testing results are available is known as definitive therapy [14]. The timely initiation of effective antimicrobial treatment based on susceptibility testing results within 72 h of infection is considered early definitive therapy, whereas treatments that do not meet this time requirement are considered delayed definitive therapy [15]. Combination therapy refers to the use of multiple in vitro active antimicrobial treatments. A Short treatment duration was characterized by the administration of in vitro active antimicrobial treatment for less than 10 days, whereas a long treatment duration referred to the administration of such treatment for 10 days or longer [16]. Post-ERCP pancreatitis (PEP) was identified when the serum amylase level increased to more than three times the usual limit, along with prolonged abdominal discomfort lasting more than 24 h after ERCP [17]. Malignant biliary strictures were identified when biliary strictures were induced by malignancies. A biliary leak was recognized when bile leaked from any of the ducts channeling bile to the small intestine [18]. Instances of an abnormal connection between the epithelial surface of the pancreatic duct and another surface were used to define a pancreatic fistula [19]. Hypoproteinaemia was identified when the serum albumin level was less than 30 g/L on the same day (or within 24 h) that a positive CRE blood culture sample was obtained.

### Tests for identifying bacteria and determining their sensitivity to drugs

The process of isolating and identifying pathogenic bacteria was conducted in strict adherence to the stipulations outlined in the National Clinical Laboratory Procedures. Cultures derived from clinical specimens were scrutinized for identification and susceptibility via the automated VITEK2 system (BioMérieux, France). Drug resistance was determined via both the Kirby–Bauer (K-B) method (disk diffusion method) and broth microdilution (BMD), where the BMD was utilized to determine the minimum inhibitory concentration (MIC). The cutoffs set by the European Committee on Antimicrobial Susceptibility Testing (EUCAST) were used for the antibiotics tigecycline and colistin, whereas the interpretation of the other antibiotics adhered to the standards specified in the Clinical and Laboratory Standards Institute (CLSI) document [20, 21].

### Data collection and variable analysis

Our database included 36 clinical variables. Categorical variables are presented as percentages and numerical values, and comparisons were made via either the chi-square test or Fisher’s exact test. Continuous variables were compared via the independent t test or the Mann‒Whitney U test. The significance threshold was set at a p value less than 0.05.

### Identification of significant variables

To identify the key characteristics, we used the least absolute shrinkage and selection operator (LASSO) regression model, which selects variables with nonzero coefficients. Univariate Cox regression analysis was conducted to analyze the study outcomes, comparing the survival and nonsurvival cohorts. Hazard ratios (HRs) and 95% confidence intervals (CIs) were calculated for each variable. Variables that were significant in the univariate analysis were subsequently included in the multivariate Cox regression to identify independent risk factors influencing the outcome. These factors are presented as HRs with 95% CIs and p values.

### Development of the nomogram

Based on the multivariate Cox regression analysis, we developed a nomogram to predict the risk of 30-day mortality. The performance of the nomogram was evaluated by calibrating the model via bootstrapping with 1,000 samples and by calculating the C-index.

### Validation and clinical usability

To validate the nomogram, we compared its performance with the SOFA score and logistic organ dysfunction score (LODS) via receiver operating characteristic (ROC) curve analysis and decision curve analysis (DCA). X-tile software was used to determine the optimal threshold for categorizing patients into low-risk and high-risk groups. The Kaplan‒Meier method was used to estimate cumulative survival rates over time. A p value of less than 0.05 was considered statistically significant. All the statistical analyses were performed via STATA 15.1 (College Station, Texas) and R 3.6.2 (Chicago, Illinois) software.

## Results

### Patient characteristics

**Fig. 1:**
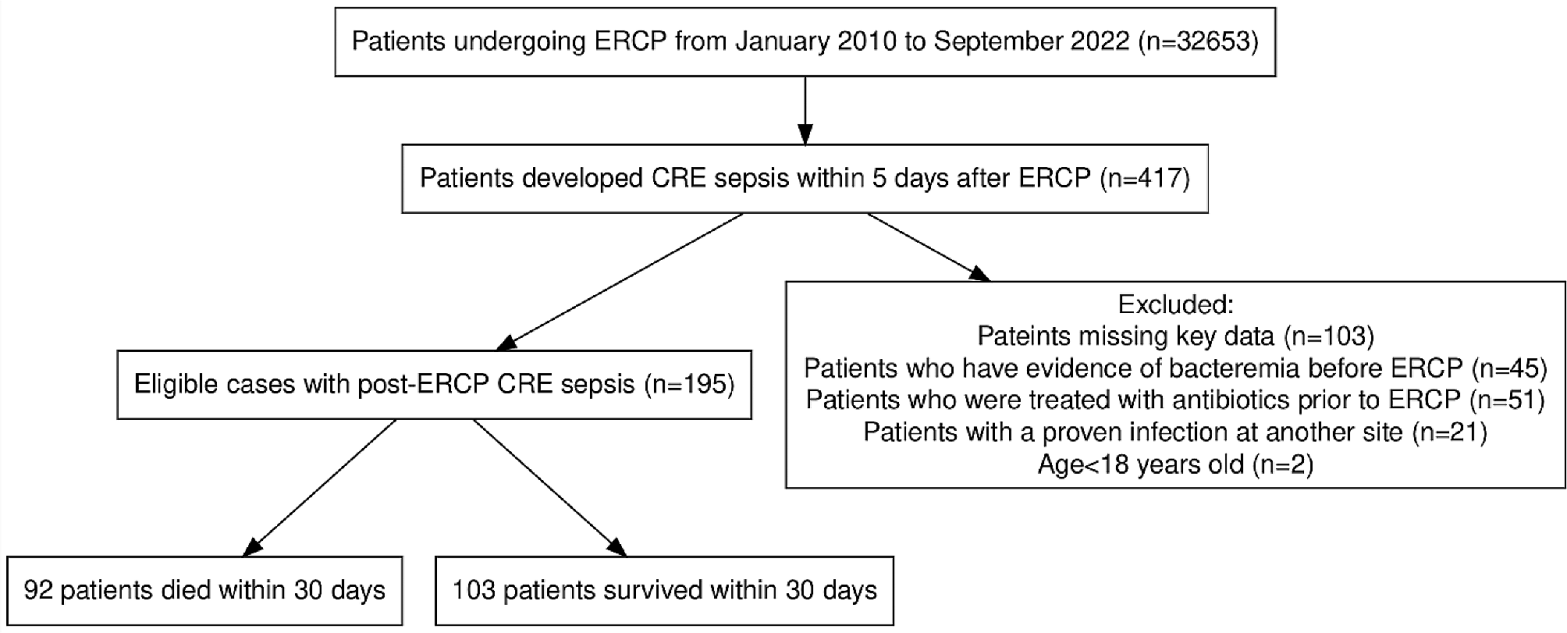
Flowchart delineating the inclusion of patients with CRE sepsis following ERCP. Abbreviations: ERCP, Endoscopic retrograde cholangiopancreatography; CRE, Carbapenem-resistant *Enterobacterales*

During the specified study interval, 417 patients developed CRE sepsis within 5 days post-ERCP. After applying the inclusion and exclusion criteria, a total of 195 patients were chosen for the present study. The study flow chart is shown in Fig. 1. The patients were divided into two groups: (1) the survivor group (*n* = 103), which included individuals who survived for more than 30 days after the onset of post-ERCP CRE sepsis, and (2) the nonsurvivor group (*n* = 92), which included individuals who died within 30 days after the onset of post-ERCP CRE sepsis. Table 1 shows the baseline characteristics of these groups. Categorical variables were compared via the chi-square test or Fisher’s exact test. Significant differences between the survivor and nonsurvivor groups were observed in terms of the percentages of patients aged > 80 years (10.7% and 27.2%, respectively; *p* < 0.01), ICU admission within 90 days prior to ERCP (4.9% and 16.3%, respectively; *p* < 0.01), hypoproteinemia (51.5% and 81.5%, respectively; *p* < 0.01), quick Pitt bacteremia score ≥ 2 (34.0% and 75.0%, respectively; *p* < 0.01), cholangioscopy (4.9% and 14.1%, respectively; *p* = 0.03), PEP (3.9% and 12%, respectively; *p* = 0.03), post-ERCP perforation (2.9% and 13%, respectively; *p* = 0.01), inappropriate empirical therapy (11.7% and 48.9%, respectively; *p* = 0.01), delayed definitive therapy (8.7% and 20.7%, respectively; *p* = 0.02), and short treatment duration (< 10 days) (24.3% and 41.3%, respectively; *p* = 0.01).

**Table 1:** Basic clinical characteristics of Post ERCP patients with CRE sepsis

Variables	Total	Survivor	Death	Test statistic OR (95%CI), *P* value
	*N* = 195	*N* = 103	*N* = 92	
**Patients conditions**				
Age (years, mean ± standard deviation) Age > 80 (No.%) Male sex (No.%) Charlson comorbidity index > 4 (No.%)	71.1 ± 12.0 36 (18.5) 135 (69.2) 96 (49.2)	68.2 ± 12.8 11 (10.7) 70 (68.0) 50 (48.5)	74.4 ± 10.3 25 (27.2) 65 (70.7) 46 (50.0)	**1.1(1.0-1.1)**, ***P*** < 0.01 **3.1(1.4–6.8)**, *P* < 0.01 1.1(0.6–2.1), *P* = 0.68 1.1(0.6–1.9), *P* = 0.84
**Exposures within 90 days before ERCP**				
Antibiotics (No.%) Prior hospitalization (No.%) Invasive procedures (No.%) ICU admission (No.%)	62 (31.8) 29 (14.9) 48 (24.6) 20 (10.3)	33 (32.0) 16 (15.5) 25 (24.3) 5 (4.9)	29 (31.5) 13 (14.1) 23 (25.0) 15 (16.3)	0.9(0.5–1.8), *P* = 0.94 0.8(0.4–1.9), *P* = 0.78 1.0(0.5–1.9), *P* = 0.91 **3.8(1.3–10.9)**, **P < 0.01**
**Exposures within 30 days before ERCP**				
Immunosuppressive agents (No.%)	19 (9.7)	10 (9.7)	9 (9.8)	0.9(0.3–2.6), *P* = 0.99
**Epidemiology**				
Time from ERCP to sepsis < 2 days (No.%)	34 (17.4)	13 (12.6)	21 (22.8)	0.4(0.2-1.0), *P* = 0.06
**Comorbidities**				
Prior CRE infection history (No.%) Cerebrovascular diseases (No.%) Malignant tumor (No.%) Diabetes (No.%) Cirrhosis (No.%) Hypoproteinemia (No.%)	67 (34.4) 23 (11.8) 64 (32.8) 28 (14.4) 17 (8.7) 128 (65.6)	35 (34.0) 12 (11.7) 33 (32.0) 15 (14.6) 9 (8.7) 53 (51.5)	32 (34.8) 11 (12.0) 31 (33.7) 13 (14.1) 8 (8.7) 75 (81.5)	0.9(0.5–1.7), *P* = 0.91 1.0(0.4–2.5), *P* = 0.95 0.9(0.5–1.7), *P* = 0.81 0.9(0.4–2.2), *P* = 0.93 0.9(0.3–2.7), *P* = 0.99 **4.2(2.2–7.9)**, *P* < 0.01
**Illness severity at time of CRE sepsis**				
qPitt score ≥ 2 (No.%) APACHE II score > 20 (No.%)	104 (53.3) 17 (8.7)	35 (34.0) 8 (7.8)	69 (75.0) 9 (9.8)	**5.8(3.1–10.8)**, *P* < 0.01 1.3(0.5–3.5), *P* = 0.62
**Indication for ERCP**				
Malignant biliary stricture (No.%) Benign biliary stricture (No.%) Bile duct stone (No.%) Pancreatic duct stone (No.%) Pancreatic duct stricture (No.%) Bile leak (No.%) Pancreatic fistula (No.%)	60 (30.8) 31 (15.9) 43 (22.1) 23 (11.8) 167 (85.6) 5 (2.6) 4 (2.1)	31 (30.1) 16 (15.5) 23 (22.3) 12 (11.7) 85 (82.5) 3 (2.9) 2 (1.9)	29 (31.5) 15 (16.3) 20 (21.7) 11 (12.0) 82 (89.1) 2 (2.2) 2 (2.2)	1.1(0.6–1.9), *P* = 0.83 1.1(0.5–2.3), *P* = 0.88 0.9(0.5–1.9), *P* = 0.92 0.9(0.4–2.3), *P* = 0.95 0.9(0.4–1.9), *P* = 0.78 0.7(0.1–4.5), *P* = 0.74 1.1(0.2–8.1), *P* = 0.91
**Procedure-related**				
Biliary stent placement (No.%) Cholangioscopy (No.%) Biliary sphincterotomy (No.%) Bile duct stone removal (No.%) Bilde duct radiofrequency ablation (No.%) Total duration > 45 min (No.%) Post ERCP pancreatitis (No.%) Post ERCP perforation (No.%) Post ERCP bleeding (No.%)	167 (85.6) 18 (9.2) 91 (46.7) 37 (19.0) 31 (15.9) 21 (10.8) 15 (7.7) 15 (7.7) 10 (5.1)	85 (82.5) 5 (4.9) 46 (44.7) 19 (18.4) 16 (15.5) 11 (10.7) 4 (3.9) 3 (2.9) 5 (4.9)	82 (89.1) 13 (14.1) 45 (48.9) 18 (19.6) 15 (16.3) 10 (10.9) 11 (12.0) 12 (13.0) 5 (5.4)	0.6(0.3–1.3), *P* = 0.19 **3.2 (1.1–9.4)**, *P* = 0.03 0.8(0.5–1.5), *P* = 0.55 1.1(0.5–2.2), *P* = 0.84 1.1(0.5–2.3), *P* = 0.88 1.0(0.4–2.5), *P* = 0.97 **3.4(1.0-10.9)**, *P* = 0.03 **5.0(1.3–18.3)**, *P* = 0.01 1.1(0.3-4.0), *P* = 0.85
**Antibiotic Antimicrobial treatment**				
Inappropriate empirical therapy (No.%) Non-early-appropriate therapy (No.%) Short-duration < 10 days (No.%)	57 (29.2) 28 (14.4) 66 (33.8)	12 (11.7) 9 (8.7) 25 (24.3)	45 (48.9) 19 (20.7) 38 (41.3)	**7.3(3.5–15.0)**, *P* < 0.01 **2.7(1.2–6.3)**, *P* = 0.02 **2.2(1.2–4.1)**, *P* = 0.01

Note: *P < 0.05 (bold values) was considered statistically significant

Abbreviations: ERCP, Endoscopic retrograde cholangiopancreatography; CRE, Carbapenem-resistant Enterobacterales; qPitt, A quick version of the Pitt Bacteremia Score; APACHE II, Acute Physiology and Chronic Health Evaluation II; ICU, intensive care unit; OR, Odds Ratio; CI, Confidence Interval

### LASSO regression analysis

**Fig. 2:**
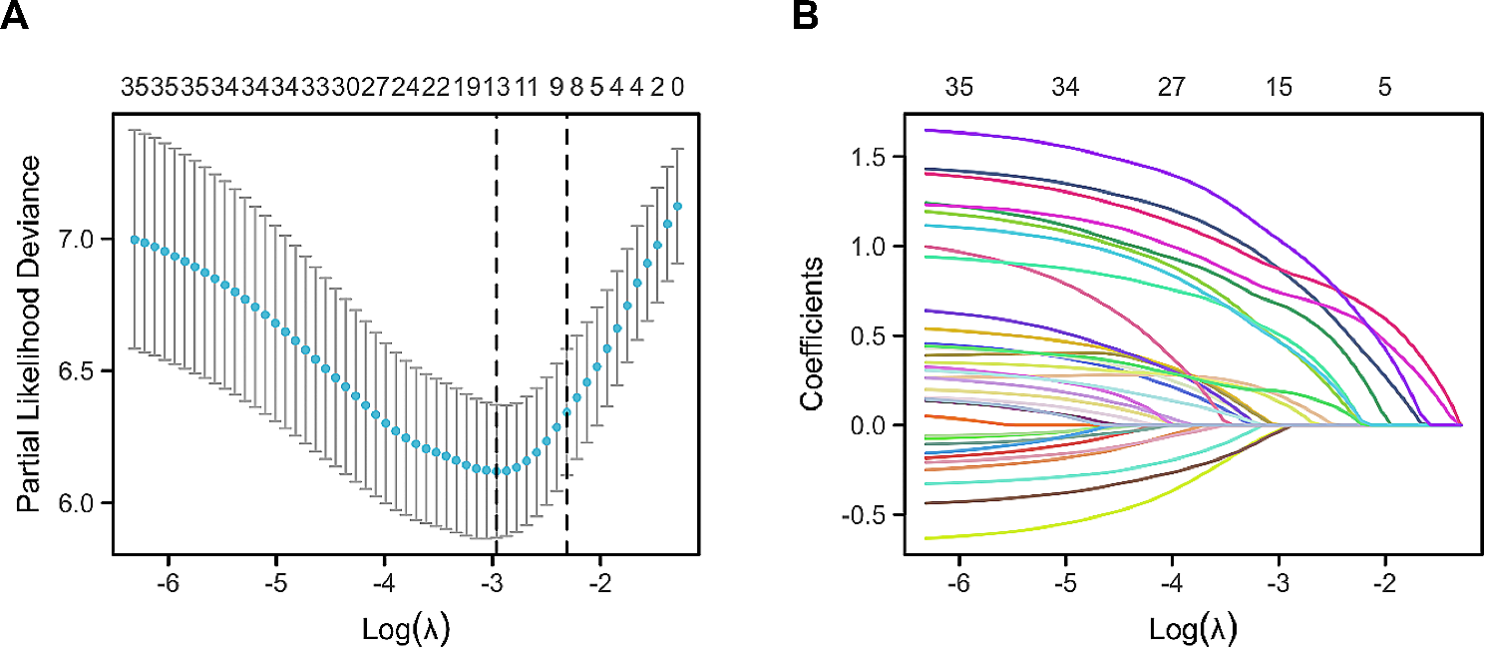
LASSO regression variable selection. (**A**) The variation attributes of the variable coefficients; (**B**) the selection procedure for the optimal value of the parameter λ within the LASSO regression model

Initially, a total of 36 relevant factors were combined into the LASSO regression model to identify potential predictors. Thirteen possible factors with coefficients greater than zero were identified, as shown in Fig. 2A. These factors included age > 80 years, hospitalization within 90 days prior to ERCP, ICU admission within 90 days prior to ERCP, CRE sepsis within 2 days after ERCP, diabetes, hypoproteinemia, quick Pitt bacteremia score ≥ 2, cholangioscopy, PEP, post-ERCP perforation, inappropriate empirical therapy, delayed definitive therapy, and short treatment duration (< 10 days). Figure 1B shows the alterations in the LASSO coefficients.

### Risk factors for mortality

Table 2 shows the 13 predictors identified via LASSO regression analysis. These predictors were then further examined via both univariate and multivariate Cox regression analyses. In the multivariate analysis, eight factors were identified as significant predictors of mortality within a 30-day period following post-ERCP CRE sepsis: age > 80 years (HR 2.61; 95% CI 1.53–4.47; *p* < 0.001), ICU admission within 90 days prior to ERCP (HR 2.64; 95% CI 1.39–5.04; *p* = 0.003), hypoproteinemia (HR 4.55; 95% CI 2.48–8.34; *p* < 0.001), quick Pitt bacteremia score ≥ 2 (HR 2.61; 95% CI 1.55–4.37; *p* < 0.001), PEP (HR 2.52; 95% CI 1.29–4.92; *p* = 0.007), inappropriate empirical therapy (HR 3.48; 95% CI 2.19–5.53; *p* < 0.001), delayed definitive therapy (HR 2.64; 95% CI 1.52–4.60; *p* < 0.001), and short treatment duration (< 10 days) (HR 5.03; 95% CI 2.97–8.52; *p* < 0.001).

**Table 2:** Univariate and multivariate COX regression analysis of predictors of all-cause 30 day mortality patients with CRE sepsis post ERCP.

	Univariable				Multivariable		
Characteristic	HR	95% CI	*p*-value	HR		95% CI	*p*-value
Age > 80	1.88	1.19–2.98	**0.007**	2.61		1.53–4.47	**< 0.001**
Prior hospitalization within 90 days	0.93	0.52–1.67	0.800	0.69		0.36–1.30	0.246
ICU admission within 90 days	2.45	1.40–4.26	**0.002**	2.64		1.39–5.04	**0.003**
Time from ERCP to Sepsis < 2 days	1.63	1.00-2.65	**0.050**	1.50		0.87–2.56	0.143
Diabetes	0.90	0.50–1.62	0.732	0.70		0.37–1.32	0.272
Hypoproteinemia	2.86	1.69–4.86	**< 0.001**	4.55		2.48–8.34	**< 0.001**
Quick Pitt Bacteremia Score ≥ 2	3.53	2.20–5.67	**< 0.001**	2.61		1.55–4.37	**< 0.001**
Cholangioscope	2.11	1.17–3.80	**0.013**	1.48		0.80–2.75	0.211
Post ERCP pancreatitis	2.19	1.16–4.12	**0.015**	2.52		1.29–4.92	**0.007**
Post ERCP perforation	2.98	1.62–5.48	**< 0.001**	1.24		0.60–2.55	0.563
Inappropriate Empirical therapy	3.60	2.38–5.45	**< 0.001**	3.48		2.19–5.53	**< 0.001**
Non-early-appropriate therapy	1.89	1.14–3.13	**0.014**	2.64		1.52–4.60	**< 0.001**
Short Duration < 10 days	2.49	1.64–3.78	**< 0.001**	5.03		2.97–8.52	**< 0.001**

Note: *P < 0.05 (bold values) was considered statistically significant.

**Abbreviations**: HR = Hazard Ratio; CI = Confidence Interval; CRE, Carbapenem-resistant Enterobacterales; ERCP, Endoscopic retrograde cholangiopancreatography

### Creation of the nomogram for predicting mortality within 30 days

**Fig. 3:**
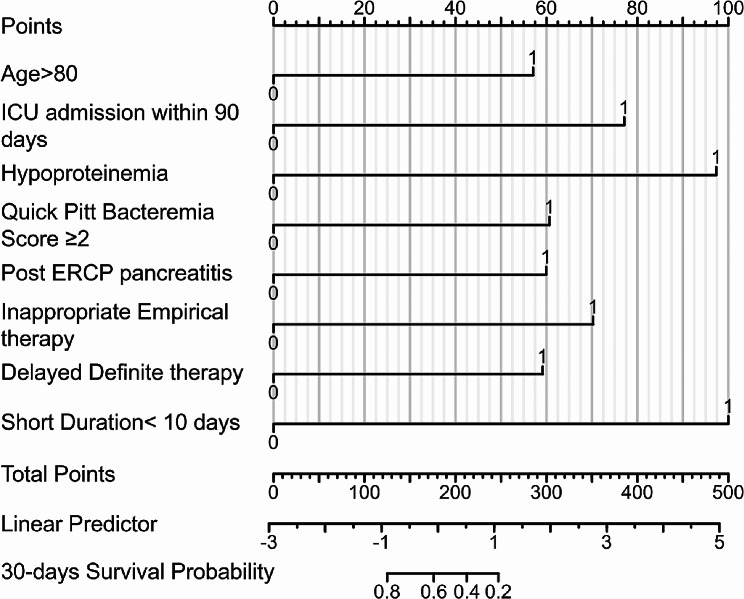
Estimating the likelihood of 30-day mortality in patients with CRE sepsis post-ERCP: a model utilizing nomogram predictions. Abbreviations: ERCP, Endoscopic retrograde cholangiopancreatography; CRE, Carbapenem-resistant *Enterobacterales*

A clinical chart was subsequently created using the significant predictors identified via multivariate Cox regression analysis, as these predictors were observed to greatly impact the clinical results (Fig. 3). In the nomogram, every predictor was visually depicted and assigned a corresponding score. Aggregating the scores of each predictor, which correspond to the predicted probability of the clinical event, enables the calculation of the cumulative total points indicating a clinical event.

### Assessment and validation of the nomogram

**Fig. 4:**
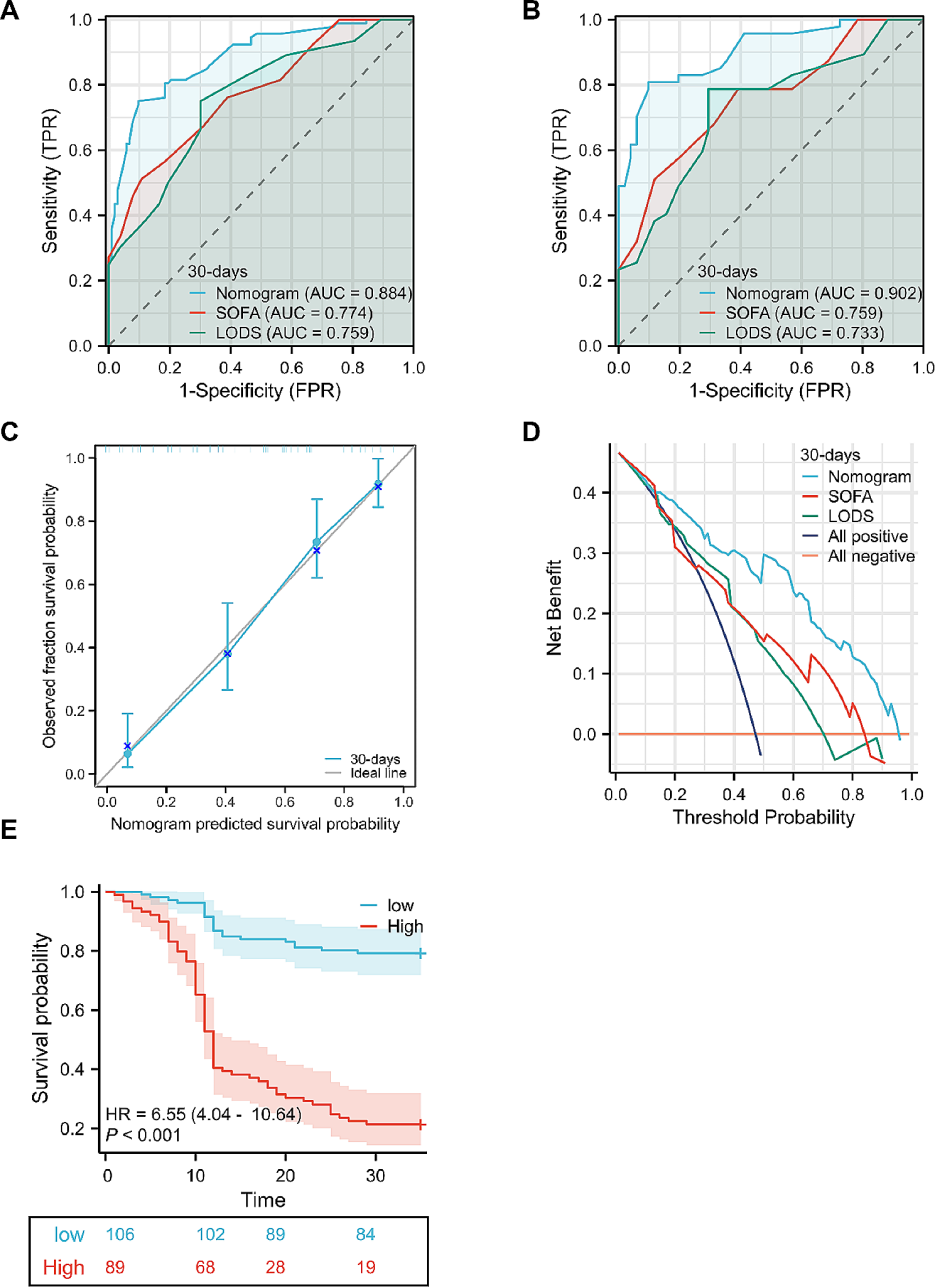
Assessment and verification of the nomogram. (**A**) ROC curve representation of the nomogram, SOFA score, and LODS score in the training set and (**B**) internal validation set. (**C**) Construction of calibration curves in the training set. (**D**) DCA curve depicting medical intervention efficacy in patients as evaluated by the nomogram, SOFA score, and LODS. (**E**) Kaplan‒Meier survival curves for patients with CRE sepsis post-ERCP grouped according to the nomogram. The p value (< 0.001) was ascertained via the log-rank test. The information within the table shows the number at risk at particular time instances. Abbreviations: Sequential Organ Failure Assessment score (SOFA), Logistic Organ Dysfunction Score (LODS)

The developed nomogram demonstrated excellent performance in predicting the risk of 30-day mortality among patients suffering from post-ERCP CRE sepsis, as indicated by a C-index of 0.884. The strength of this model was confirmed by bootstrapping validation, which revealed a C-index of 0.902 for the cohort (Fig. 4A-B). When the nomogram was compared with the SOFA and LODS metrics, the area under the ROC curve (AUC) was significantly better. The calibration efficacy of the model was then thoroughly assessed via a calibration curve, which demonstrated excellent calibration performance (Fig. 4C). The clinical utility of the model (Fig. 4D) was assessed through DCA, which demonstrated that the nomogram model provided net benefits across a broad spectrum of threshold probabilities. Using X-tile software, the point of separation that offers the highest level of sensitivity and specificity in differentiating patients at low risk and high risk was determined. The 30-day mortality rate among post-ERCP CRE sepsis patients in the high-risk group was significantly greater than that in the low-risk group (all patients 79.2% vs. 20.8%, *p* < 0.001; HR 6.55, 95% CI 4.04–10.64) (Fig. 4E).

### Effects of different antimicrobial regimens

**Fig. 5:**
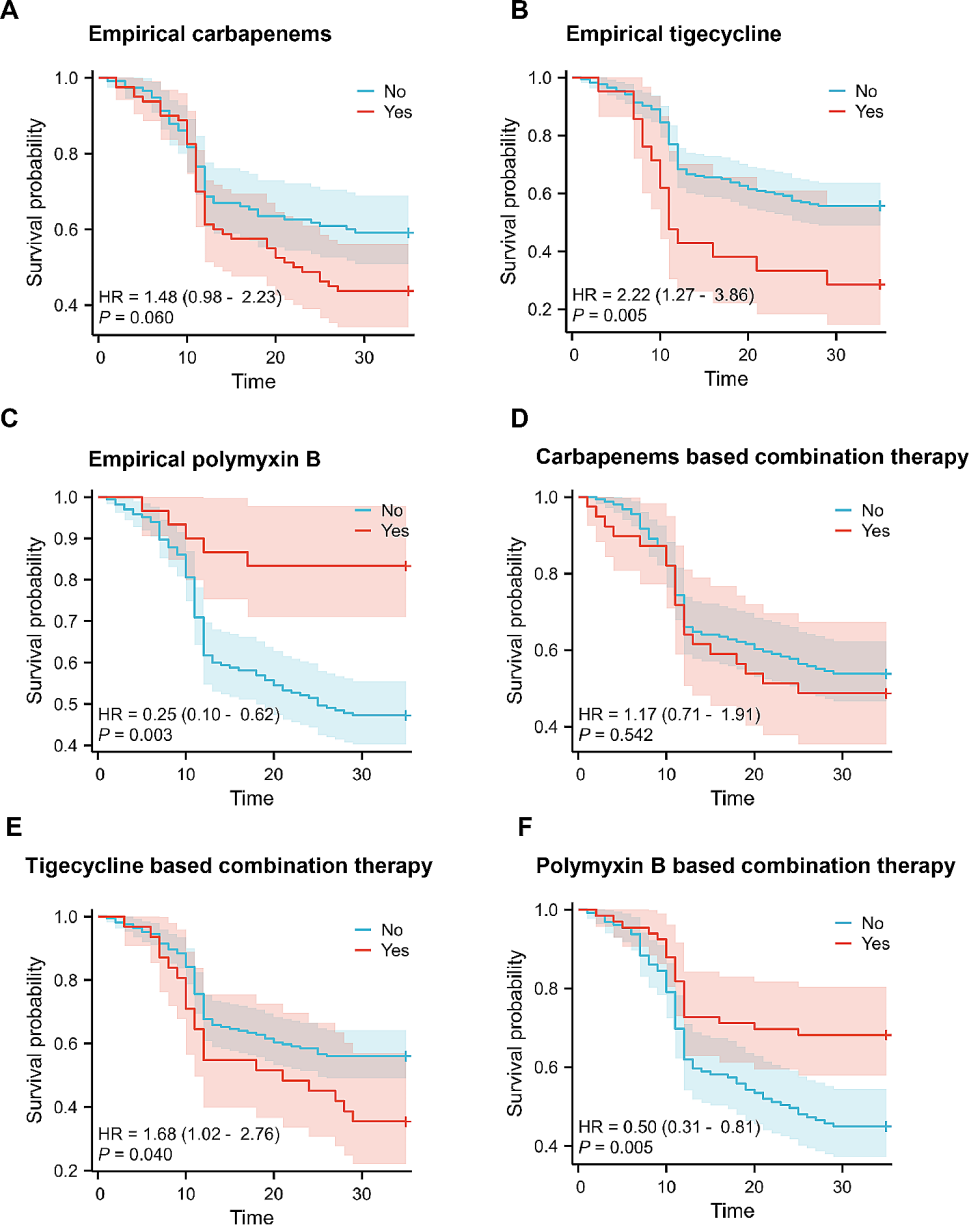
Visual representation of the consequences of different antimicrobial therapies shown through Kaplan‒Meier curves. (**A**) There was no difference in 30-day mortality among patients who were given empirical carbapenems. (**B-C**) Patients who received empirical tigecycline had a negative prognosis within 30 days, whereas those who received empirical polymyxin B had a survival benefit within the same time frame. (**D**) There was no variation in the 30-day mortality rate among patients who received combination therapy with carbapenem. (**E**) Individuals who received combination therapy involving tigecycline experienced an unfavorable prognosis within a 30-day period. (**F**) On the other hand, individuals who received combination therapy involving polymyxin B experienced a survival advantage for a period of 30 days

Different antimicrobial treatments have a wide range of clinical effectiveness, but the best antimicrobial therapy for post-ERCP CRE sepsis is still unknown. According to the Kaplan‒Meier analysis, there was no notable difference in the 30-day mortality rate among patients regardless of whether they received empirical carbapenem therapy (*p* = 0.06) (Fig. 5A). According to our dataset, empirical tigecycline treatment was associated with unfavorable outcomes (*p* = 0.005) (Fig. 5B), whereas empirical polymyxin B treatment was associated with favorable outcomes (*p* = 0.003) (Fig. 5C). Further examination was performed to assess the influence of the combined treatment. There was no noticeable variation in the 30-day mortality rate among patients regardless of whether they received carbapenem combination therapy (*p* = 0.542) (Fig. 5D). Notably, tigecycline combination treatment markedly increased 30-day mortality (*p* = 0.04) (Fig. 5E), whereas combination therapy involving polymyxin B substantially increased survival within a 30-day period (*p* = 0.005) (Fig. 5F).

## Discussion

Our study identified several key factors contributing to 30-day mortality in patients with post-ERCP CRE sepsis. The significant independent risk factors included age > 80 years, ICU admission within 90 days prior to ERCP, hypoproteinemia, quick Pitt bacteremia score ≥ 2, post-ERCP pancreatitis (PEP), inappropriate empirical therapy, delayed definitive therapy, and short treatment duration (< 10 days). These variables were used to develop a nomogram for predicting the risk of 30-day mortality. This nomogram demonstrated strong differentiation, strong calibration, and a high C-index. Our investigation reported a 30-day all-cause mortality rate of 47.1% for post-ERCP CRE sepsis patients, with those in the high-risk group having a significantly higher mortality rate (HR 6.55).

Age > 80 years was an independent risk factor for mortality. Elderly patients often have multiple comorbidities, such as a weakened immune system and reduced organ function, which make them more susceptible to severe outcomes from CRE sepsis [22–24]. This finding underscores the importance of tailored infection prevention strategies for elderly patients, especially in the context of increasing antibiotic resistance. ICU admission within the prior 90 days also emerged as a significant risk factor. ICU patients are often critically ill and may have compromised immune responses, increasing their susceptibility to severe infections [25]. Moreover, ICU environments are hotspots for multidrug-resistant pathogens because of the frequent use of broad-spectrum antibiotics and invasive procedures [26]. This highlights the need for stringent infection control measures during ERCP for patients recently discharged from ICUs. Hypoproteinaemia is another independent risk factor, reflecting its role in indicating malnutrition and compromised immune function [27]. Low serum albumin levels can impair vascular integrity and promote bacterial invasion, exacerbating infection severity [28]. These findings underscore the multifaceted role of albumin in patient outcomes during severe infections, such as post-ERCP CRE sepsis.

A quick Pitt bacteremia score ≥ 2 was a significant predictor of poor outcomes. This score, which is designed to assess the severity of bloodstream infections, indicates substantial systemic infection and the need for intensive medical interventions [29]. Higher scores correlate with an increased risk of complications such as septic shock and organ dysfunction, which aligns with our findings. Our findings are consistent with previous research showing that the quick Pitt bacteremia score effectively predicts 30-day mortality, not only in patients with bacteremia but also in those with *K. pneumoniae* infections [30]. Clinicians should accurately calculate and interpret these scores to identify at-risk patients promptly. Early recognition allows for more intensive treatment, increased vigilance, and the potential for more aggressive or personalized therapeutic interventions. PEP was also identified as a risk factor for mortality, highlighting the importance of preventative measures during ERCP [31]. The inflamed pancreatic environment can facilitate bacterial translocation, leading to systemic infection. Patients with PEP are more susceptible to severe outcomes, emphasizing the need for careful patient management post-ERCP [32]. On the basis of our findings, clinicians should be especially vigilant in managing post-ERCP patients who develop pancreatitis, as these patients are more susceptible to severe outcomes from CRE sepsis.

Our study revealed that delayed definitive therapy and a treatment duration of less than 10 days were independent factors negatively affecting 30-day survival rates. Timely administration of appropriate antimicrobial treatment is crucial. Delayed antibiotic therapy increases mortality risk with each hour of delay [33]. Starting appropriate treatment within the first 24 h after blood culture is most beneficial, whereas delays beyond 24 h significantly increase mortality [34, 35]. Timely empirical treatment is therefore essential. Patients receiving appropriate empirical treatment had better outcomes, which is consistent with the findings of previous studies. However, the optimal treatment for CRE sepsis remains unclear. Our study revealed higher mortality with empirical tigecycline use, likely due to its bacteriostatic nature and limited efficacy against *Pseudomonas aeruginosa* [36]. Conversely, polymyxin B has shown survival benefits, demonstrating efficacy against multidrug-resistant gram-negative bacteria [37], favorable pharmacokinetics, and a reduced risk of kidney damage [38]. A Japanese multicenter study also supported the effectiveness of polymyxin B in reducing mortality in sepsis patients [39]. Combination therapy, particularly polymyxin B, provides a 30-day survival advantage [40, 41]. A short treatment duration (< 10 days) was a risk factor for 30-day mortality, likely due to inadequate bacterial eradication, leading to persistent infections. Prolonged therapy (≥ 14 days) results in better outcomes [42]. The rapid onset of CRE sepsis within 5 days post-ERCP indicates a complex etiology, possibly involving contaminated duodenoscopes and endogenous bacteria entering the bloodstream during the procedure. Further research is necessary to understand these factors and develop effective preventive measures.

We developed a validated tool to predict the 30-day mortality risk for patients with post-ERCP CRE sepsis. This tool helps healthcare professionals identify high-risk patients early, facilitating initial risk categorization and personalized treatment. Fundamentally, this nomogram has the potential to improve patient outcomes and enhance clinical decision-making in managing post-ERCP CRE sepsis.

### Limitations

We acknowledge several limitations within our study. The generalizability of our findings is limited, as the data were collected exclusively from a patient cohort in a tertiary hospital in Zhejiang Province, which may not represent the wider range of Chinese patients. Furthermore, our examination did not cover every possible variable affecting the 30-day mortality rate. We were unable to thoroughly examine numerous potential factors that could affect the risk of 30-day mortality, such as specific strains of CRE and variations in enzyme types, owing to the inherent limitations of our research environment. Despite our thorough examination of the strength of the nomogram via bootstrapping, the lack of external validation raises doubts about the generalizability of the results to different populations in various regions and countries. This underscores the necessity for subsequent external validation within a more expansive patient population to further ascertain the applicability and validity of the nomogram in different clinical settings and geographic locations.

## Conclusions

In this study, risk factors for 30-day mortality in patients with CRE sepsis following ERCP were successfully identified, and a validated nomogram was developed to accurately predict this risk. Nomograms are tools that clinicians can use to quickly identify patients at high risk, thus facilitating timely and appropriate interventions against CRE sepsis. Further research is needed to confirm whether the nomogram developed herein can be used to guide personalized treatments can decrease mortality rates and improve outcomes in these patients. External validation of the nomogram is also essential to ensure its effectiveness across different healthcare settings.

## Data Availability

The datasets used in this study can be obtained from the corresponding author upon reasonable request.
